# Systematic Investigation of the Degradation Properties of Nitrile-Butadiene Rubber/Polyamide Elastomer/Single-Walled Carbon Nanotube Composites in Thermo-Oxidative and Hot Oil Environments

**DOI:** 10.3390/polym16020226

**Published:** 2024-01-12

**Authors:** Guangyong Liu, Huiyu Wang, Tianli Ren, Yuwei Chen, Susu Liu

**Affiliations:** 1 Key Laboratory of Rubber-Plastics of Ministry of Education, Qingdao University of Science & Technology, Qingdao 266042, China; liuguangyong@qust.edu.cn (G.L.); 15666426387@163.com (H.W.); yuweichen@qust.edu.cn (Y.C.); 2 Mississippi Polymer Institute, University of Southern Mississippi, Hattiesburg, MS 39406, USA; tianli.ren@usm.edu

**Keywords:** nitrile-butadiene rubber, polyamide elastomer, single-walled carbon nanotubes, composite, swelling, aging

## Abstract

The physical blending method was used in order to prepare nitrile-butadiene rubber/polyamide elastomer/single-walled carbon nanotube (NBR/PAE/SWCNT) composites with better thermal-oxidative aging resistance. The interactions between SWCNTs and NBR/PAE were characterized using the Moving Die Rheometer 2000 (MDR 2000), rheological behavior tests, the equilibrium swelling method, and mechanical property tests. The 100% constant tensile stress and hardness of NBR/PAE/SWCNT composites increased from 2.59 MPa to 4.14 MPa and from 62 Shore A to 69 Shore A, respectively, and the elongation decreased from 421% to 355% with increasing SWCNT content. NBR/PAE/SWCNT composites had improved thermal-oxidative aging resistance due to better interactions between SWCNTs and NBR/PAE. During the aging process, the tensile strength and elongation at break decreased with the increase in aging time compared to the unaged samples, and the constant tensile stress gradually increased. There was a more significant difference in the degradation of mechanical properties when aged in a variety of oils. The 100% constant tensile stress of NBR/PAE/SWCNT composites aged in IRM 903 gradually increased with aging time while it gradually decreased in biodiesel. The swelling index gradually increased with increasing SWCNT content. Interestingly, the swelling index of the composites in cyclohexanone decreased with the increase in SWCNT content. The reasons leading to different swelling behaviors when immersed in different kinds of liquids were investigated using the Hansen solubility parameter (HSP) method, which provides an excellent guide for the application of some oil-resistant products.

## 1. Introduction

Nitrile butadiene rubber (NBR) is an organic polymer elastic compound with superior abrasion resistance, processability, and excellent oil resistance [1,2,3,4]. Hence, NBR materials are widely used in the manufacture of some seals, fuel hoses, and other oilfield rubber products [5,6,7,8,9]. Generally, in real conditions, NBR seals are frequently used in severe applications, so the degradation of NBR in these environments is unavoidable [10,11]. Most seal failures are caused by rubber aging, which leads to substantial economic losses. Bingqi Jiang et al. [12] investigated the effect of thermal aging in oil on the friction and wear properties of NBR. Boli et al. [13] studied NBR seals exposed to different aging atmospheres and evaluated the mechanical properties, tribological behavior, and energy dissipation of aged NBR. In the study, thermo-oxidative aging significantly degraded the mechanical properties and greatly affected the hysteresis characteristics during friction, further causing degradation.

As rubber products become more widespread in today’s society, the requirements for rubber product performance are becoming increasingly stringent. For environmental protection and sustainable development, incorporating some filler into the polymer matrix plays an ever more critical role in reducing costs and improving performance [14,15,16,17,18]. Emad S. Shafik et al. [19] used red brick waste (RBW) powder as a reinforcing filler in NBR to prepare eco-friendly composites. The findings indicated that such composites could be used for insulation and antistatic applications. In addition, magnetic measurements have shown superparamagnetic behavior in NBR/RBW composites. Dawei Tang et al. [20] studied the incorporation of waste brick powder as a filler in SBR to enhance its mechanical properties. This was performed to reduce the polymer product’s cost. Xumin Zhang et al. [21] reported that a novel filler, graphene oxide, enhanced the compatibility and mechanical properties of carboxylated nitrile butadiene rubber/styrene butadiene rubber. The results showed that the addition of graphene oxide could significantly improve the thermal and mechanical properties of the blends as well as their compatibility. Shubham C. Ambilkar et al. [22] employed three different surface modifiers, namely sodium dodecyl sulfate (SDS, a surfactant), 3-(trimethoxy silyl) propyl methacrylate (MPS, an organosilane), and tris (hydroxymethyl) aminomethane (Tris, an amine buffer), to modify the surface of sol–gel derived from in situ generated zirconia and investigated the thermal, morphological, mechanical, swelling, rheological, and dielectric properties. This study revealed that Tris could be a potential surface modifier for metal oxides like zirconia, as an alternative to organosilane, to reinforce the elastomer matrices. S. Utrera-Barrios et al. [23] introduced an innovative approach in which waste parts from toner cartridges were valorized to develop (recyclable and) self-healing elastomeric composite materials. The study found that the thermoplastic elastomers formed from high-impact polystyrene and carboxylated nitrile butadiene rubber from toner cartridge waste had high mechanical properties and self-healing ability. Moreover, an increase in toner content (up to 20 phr) resulted in an optimal balance between tensile strength and self-healing capacity. Fanghui Wang et al. [24] prepared carbon nanofibers–silica (CNFs–SiO_2_) nanocomposites by the self-assembly method, which were used as filler to obtain CNFs–SiO_2_/HNBR composites. These results indicated that CNFs–SiO_2_ as a reinforcing filler could significantly improve the rubber mechanical properties. SWCNTs exhibit an unusual one-dimensional tubular structure, high specific area, and excellent mechanical, electrical, thermal, and chemical properties [25,26,27]. It is because of these properties that SWCNTs are widely used in the fabrication of polymer composites for different applications. Kazufumi Kobashi et al. [28] proposed a method to control the dendritic structure of long carbon nanotubes (CNTs). The results showed that the electrical conductivity of carbon nanotube rubber composites could be improved by controlling the dendritic structure of CNTs. Jabulani I. Gumede et al. [29] investigated the effect of SWCNTs on the vulcanization properties and mechanical properties of recycled rubber (RR)/natural rubber (NR) blends and found that the addition of SWCNTs decreased the minimum torque, increased the scorch time as well as the vulcanization time of the RR/NR blends, and enhanced the hardness of the blends.

The effect of polyamide elastomer (PAE) on the thermal-oxidative aging resistance of NBR/PAE blends has been previously studied [30]. FTIR, DMA, SEM, and equilibrium swelling methods were used to analyze the interaction between NBR and PAE. It was found that NBR/PAE blends exhibited better tensile strength retention at high temperature. The role of PAE in improving the high-temperature aging resistance of NBR/PAE blends was also explored. Finally, the swelling behavior of rubbers in biodiesel and IRM 903 was measured. Based on earlier studies, it was found that the addition of SWCNTs resulted in the improvement of tensile strength, modulus, hardness, and electrical conductivity of the blends, while the elongation at break was slightly reduced [31,32,33,34]. Previous studies have been conducted on the effect of SWCNTs on the mechanical and electrical conductivity properties of rubber. However, no one has investigated the changes in the properties of composites containing SWCNTs during air and oil aging.

In this study, NBR/PAE/SWCNT composites with different SWCNT contents were prepared by the mechanical blending method. The interactions between SWCNTs and NBR/PAE were investigated by its vulcanization properties, using a Rubber Processing Analyzer (RPA2000), the equilibrium swelling method, and mechanical property tests. Since NBR composites are mainly used in seals, high temperatures and oil liquids can dramatically affect their properties. The degradation of mechanical properties was evaluated by performing tensile and other performance tests after thermo-oxidative aging and thermal oil aging. Moreover, the HSP method was used to explore the reasons for the changes in the swelling index of the NBR/PAE/SWCNT composites after immersion in different types of liquids, which provides an excellent guide for the study of some oil-resistant products.

## 2. Experiment

### 2.1. Materials and Preparation of NBR/PAE/SWCNT Composites

**Figure 1:**
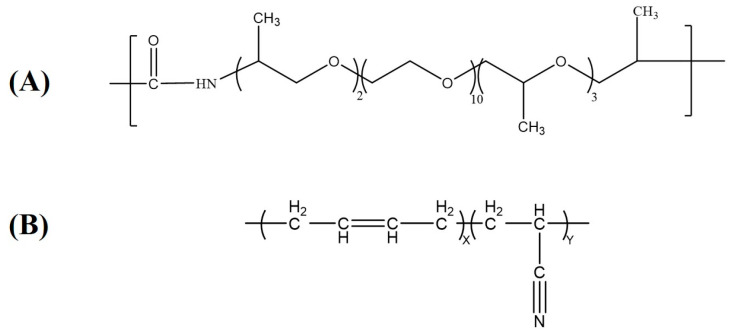
The structural formulas of (**A**) PAE of (**B**) NBR.

NBR-3445 (M_W_ = 100,000 g/mol) was purchased from Arlanxeo (The Hague, The Netherlands), with 34% acrylonitrile content. Polyamide elastomer (M_W_ = 40,000 g/mol) was supplied by Arkema France (Paris Villepinte, France), with a melting point of 170–180 °C. The curing agents and chemicals, such as sulfur, zinc oxide (ZnO), stearic acid, tetramethylthiuram disulfide (TMTD), 2,2′-dithiobis(benzothiazole)(DM), antioxidant poly (1,2-dihydro-2,2,4-trimethylquinoline) (TMQ), and *N*-1,3-dimethylbutyl-*N*′-phenyl-p-phenylenediamine (DMPPD) were supplied by Rhein Chemie, (Qingdao, China). Carbon black (N330) was produced by Cabot (Alpharetta, GA, USA). The SWCNTs were produced by OCSiAl (Novosibirsk, Russia). The used SWCNTs had a diameter of 2 nm, length of 5μm, and purity of 95%. Table 1 shows the specimen formulas. The structural formulas of NBR and PAE are shown in Figure 1.

**Figure 2:**
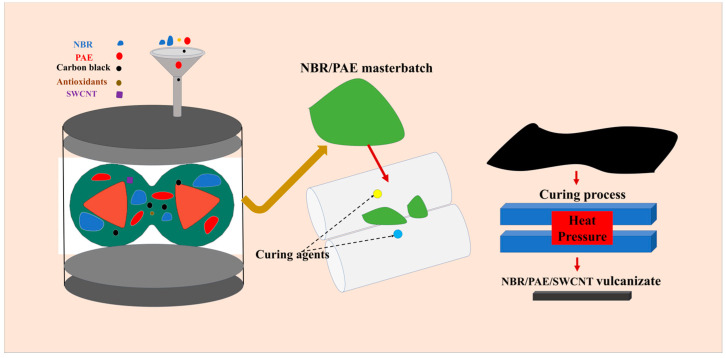
Schematic of the preparation of NBR/PAE/SWCNT composites.

The volume of the internal mixer was 300 mL. We first controlled the rotor speed of the mixer to be 60 rpm and the temperature to be 200 °C. NBR, PAE, and antioxidant were added to the mixer for 2 min to prepare the NBR/PAE masterbatch. Then, NBR/PAE masterbatch, N330, SWCNT, zinc oxide, and stearic acid were mixed in the internal mixer for 5 min at 80 °C. Finally, the roll temperature of the two-roll mill was controlled to be 30 °C. The NBR/PAE blend was firstly mixed by wrapping the rolls for 2 min, and then sulfur, accelerator DM, and TMTD were added to the two-roll mill and mixed for 8 min to make sure that the fillers were uniformly dispersed within the blend. NBR/PAE/SWCNT vulcanized rubber was prepared on a flat vulcanizer of 10 MPa at 150 °C. The preparation progress of the NBR/PAE/SWCNT composites is shown in Figure 2.

### 2.2. Experiments

Curing Characteristics. The curing characteristics, such as optimum curing time (t_90_), scorch time (t_s2_), minimum torque (M_L_), and maximum torque (M_H_), were all monitored using the Moving Die Rheometer 2000 (MDR 2000) (Akron, AL, USA) at 150 °C, according to ASTM D2084-95 [35].

Rheological behavior test. The rheological properties of the uncured and cured NBR/PAE/SWCNT composites were investigated using a Rubber Processing Analyzer (RPA 2000, Alpha Technologies, Akron, AL, USA), according to ASTM D6601 [36]. The strain sweep test was performed at 60 °C with 1 Hz frequency in the range of 0.1 to 100% strain.Scanning electron microscopy (SEM) (JSM-6700F, Japan Electronics Corp. Tokyo, Japan). To understand the morphology of the NBR/PAE/SWCNT composites, cured sheets underwent cryogenic fracture. The SEM images were recorded at an accelerating voltage of 8.0 kV.

Tensile test. The tensile testing of the NBR/PAE/SWCNT composites was conducted using a universal testing machine (Zwick/Roell Z005, Esslingen, Germany), according to ASTM D412 [37]. The samples used in the testing were dumbbell-shaped specimens. The gage length of the samples was 30 mm, and the loading speed was 500 mm/min.

Swelling index. The swelling properties of the NBR/PAE/SWCNT composites with different SWCNT contents were assessed by submerging the dry composites in cyclohexanone at room temperature and in IRM 903 and biodiesel at 115 and 135 °C for a set time.

The swelling index of all kinds of samples was calculated according to Equation (1):

(1) $$Swelling\;index=\frac{M_{f}-M_{i}}{M_{i}}\times 100$$

where *M_i_* and *M_f_* are the masses of samples before and after immersion in cyclohexanone, IRM 903, and biodiesel, respectively.

Aging process. The thermal aging process was performed at 115 and 135 °C for up to 5 days, according to ASTM D573-04 [38] The specimen was hung directly in the oven (in air) or soaked in biodiesel or IRM 903, then removed when it was finished aging and wiped for testing.

## 3. Results and Discussion

### 3.1. Curing Characteristics of the NBR/PAE/SWCNT Composites

**Figure 3:**
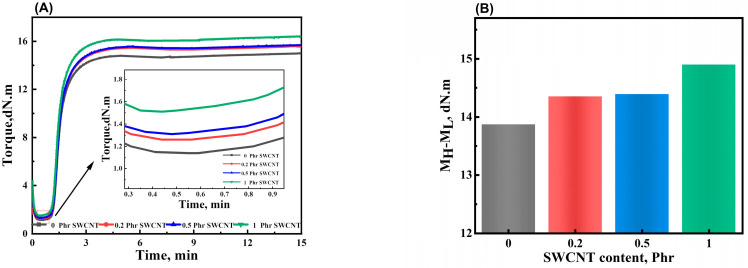
(**A**) Effect of SWCNT content on curing characteristics of NBR/PAE/SWCNT composites. (**B**) The M_H_−M_L_ values of NBR/PAE/SWCNT composites.

Figure 3A shows the vulcanization curves of the NBR/PAE/SWCNT composites with different SWCNT contents. Furthermore, Table 2 summarizes the vulcanization characteristics. It is obvious that after the vulcanization induction period, the torque of all of the samples increased gradually with time. This was due to the network formed by interactions between the rubber or between the rubber and filler. However, at the later stages of vulcanization, the torque decreased slightly. This was likely caused by the thermal cleavage reactions of the crosslinking bonds as well as the chain segments. The addition of SWCNTs caused little change in the NBR/PAE/SWCNT composite vulcanization rates. The maximum (M_H_) and minimum torque (M_L_) of the NBR/PAE/SWCNT composites increased with the increase in SWCNT content. In general, the crosslinking density of the vulcanized rubber was generally proportional to M_H_−M_L_. Figure 3B shows that the M_H_−M_L_ values of the NBR/PAE/SWCNT composites increased with higher SWCNT content. This may have been due to the high specific area of SWCNTs resulting in strong interactions with the rubber, leading to a crosslinked network in the NBR/PAE/SWCNT composites.

### 3.2. RPA 2000 Analysis of the NBR/PAE/SWCNT Composites

**Figure 4:**
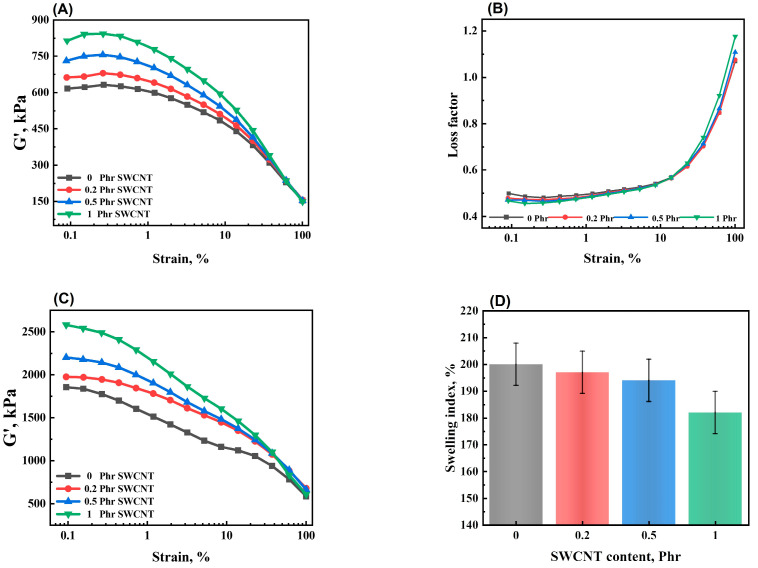
(**A**) Storage modulus (G’) and (**B**) loss factor on strain sweep for uncured NBR/PAE/SWCNT composites. (**C**) Storage modulus (G’) on strain sweep and (**D**) swelling index for cured composites.

The filler network had a significant impact on the properties of the composites. Figure 4A,B shows the storage modulus and loss factor of the uncured NBR/PAE/SWCNT composites versus strain. In summary, the difference between the initial storage modulus (G_0_) and the stabilized storage modulus (G’) indicated the size of the filler network. As observed in Figure 4A, the filler network of the NBR/PAE/SWCNT composites was gradually enhanced with increased SWCNT content. This could be attributed to the interfacial interactions between SWCNTs and NBR/PAE molecular chains, forming more restricted rubber molecular chains. Moreover, the storage modulus of the composite gradually decreased with strain increase, which was due to destruction of the filler network. Figure 4B shows that the loss factor decreased with increasing SWCNT content at low strains, while the opposite is true at high strains. This was due to the NBR/PAE/SWCNT composites having a strong filler network structure at minor strains. However, at high strains, the filler network was gradually destroyed. The interactions between SWCNTs and NBR/PAE hindered the movement of NBR/PAE molecular chains, which in turn led to an increase in the loss factor.

Figure 4C shows the strain scanning curves of vulcanized NBR/PAE/SWCNT composites. The storage modulus gradually decreased with the increase in strain. Moreover, with the increase in SWCNT content, the storage modulus of the NBR/PAE/SWCNT composites increased. This was ascribed to the fact that the interactions and mutual entanglement between SWCNTs and NBR/PAE molecular chains formed physical crosslinking points, increasing the storage modulus. In the next step, the equilibrium swelling method was employed to further investigate the effect of SWCNTs on the NBR/PAE/SWCNT composites. As shown in Figure 4D, the swelling index of the NBR/PAE/SWCNT composites decreased with the increase in SWCNT content. This further proved that SWCNTs could form a crosslinked network with the NBR/PAE molecular chains due to their high aspect ratio and interactions. This limited cyclohexanone entry into the interior of the composites and also correlated well with the results of higher M_H_-M_L_ value, storage modulus, etc.

### 3.3. Physical Properties and SEM Photographs of the NBR/PAE/SWCNT Composites

**Figure 5:**
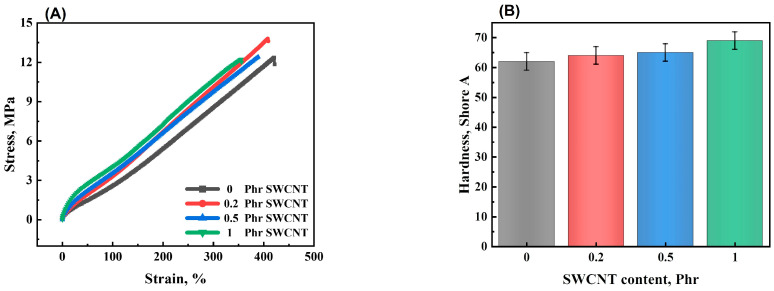
(**A**) Tensile stress–strain curves and (**B**) hardness of NBR/PAE/SWCNT composites.

**Figure 6:**
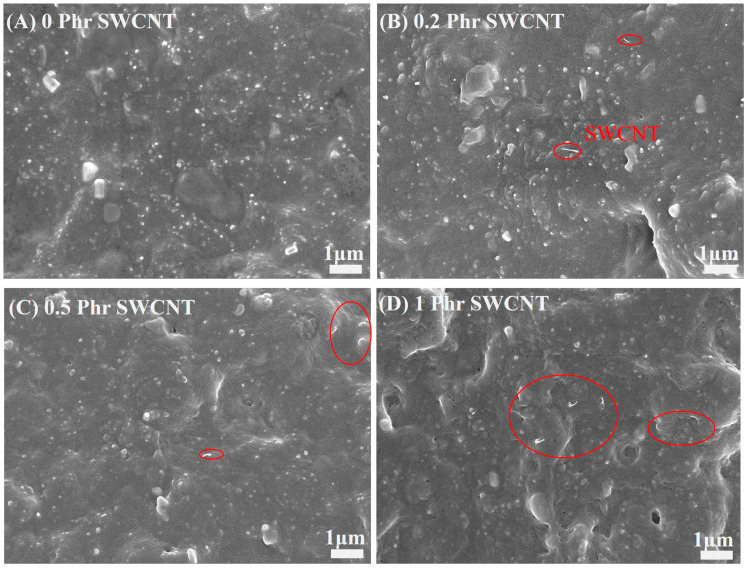
The SEM photographs of (**A**) pure NBR/PAE; (**B**) NBR/PAE-0.2SWCNT; (**C**) NBR/PAE-0.5SWCNT; (**D**) NBR/PAE-1SWCNT.

The effect of SWCNTs on the mechanical properties of the NBR/PAE/SWCNT composites was studied. Figure 5A shows the stress–strain curves of the NBR/PAE/SWCNT composites with different SWCNT contents. It was found that the 100% constant tensile stress gradually increased with the increase in SWCNT content while the elongation at break progressively decreased. In addition, the hardness of the NBR/PAE/SWCNT composites gradually increased with the increase in SWCNT content, as shown in Figure 5B, which was attributed to the higher aspect ratio and specific surface area of SWCNTs giving a better reinforcing effect to the NBR/PAE/SWCNT composites. This statement was based on Figure 6, which shows SEM images of the NBR/PAE/SWCNT composites. It can be seen that SWCNTs aggregated in the NBR/PAE/SWCNT composites. This may have acted as a stress concentrator, making the composites more susceptible to fracture.

### 3.4. The Interactions between SWCNTs and NBR/PAE of the NBR/PAE/SWCNT Composites

The interactions between SWCNTs and NBR/PAE were explored using the Hansen solubility parameter method (HSP) method. The polymer swelling behavior and polymer–solvent and polymer–polymer interactions can be predicted using the HSP method [39,40]. Herein, the energy difference (*Ra*), defined as the spatial distance between polymer and solvent and calculated by Equation (2), was introduced. Three *δ^S^* values refer to the HSPs of the solvent, and three *δ^P^* values refer to the HSPs of the rubber.

(2) $$Ra=\left[4{\left(\delta_{d}^{S}-\delta_{d}^{P}\right)}^{2}+{\left(\delta_{p}^{S}-\delta_{p}^{P}\right)}^{2}+{\left(\delta_{h}^{S}-\delta_{h}^{P}\right)}^{2}\right]$$

**Figure 7:**
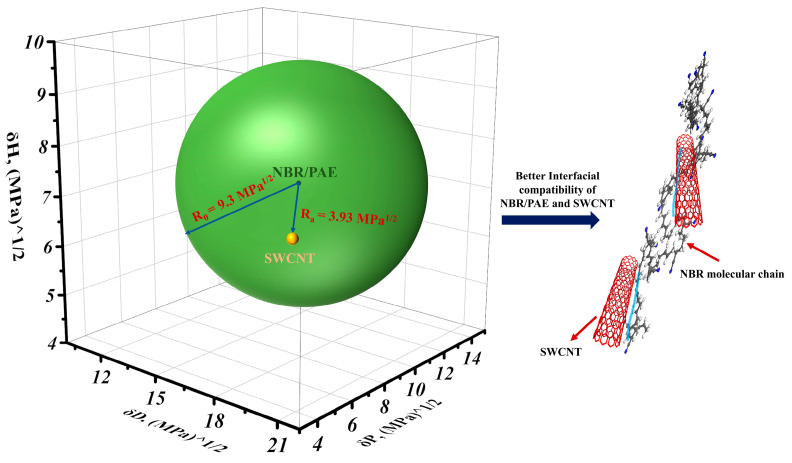
Schematic diagram of the interactions between SWCNTs and NBR/PAE.

The interactions between the rubber and solvent can be expressed in terms of their spatial distance (*Ra*), and *Ra* was calculated to be 3.9 MPa^0.5^ according to Equation (2). In accordance with our previous study, the experimentally-determined radius of the solubility sphere of NBR/PAE was 9.3 MPa^0.5^. The *Ra*/*Ro* (Figure 7) ratio is called the RED number, which reflects the relative energy difference. RED numbers less than 1.0 indicate high affinity, and progressively higher RED numbers indicate progressively lower affinity [41,42,43]. The RED between SWCNTs and NBR/PAE was calculated to be 0.42 using Equation (3). This indicated that SWCNTs had better interfacial interactions with NBR/PAE, which further limited the swelling of the NBR/PAE/SWCNT composites. This corresponded to the results of the swelling experiments.

(3) $$RED=\frac{Ra}{Ro}$$

**Figure 8:**
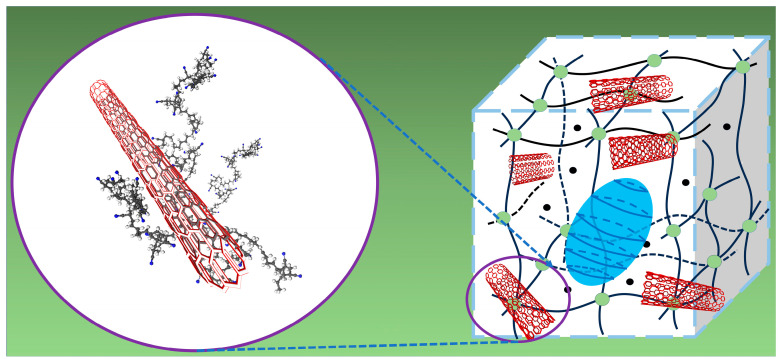
The reinforcement mechanism of SWCNT-reinforced NBR/PAE/SWCNT composites.

Considering the above test results, the reinforcement mechanism of the SWCNT-reinforced NBR/PAE/SWCNT composites is proposed in Figure 8. Because of the higher aspect ratio and specific surface area of SWCNTs, the interactions between SWCNTs and the NBR/PAE matrix resulted in better reinforcement.

### 3.5. Mechanical Properties before and after Thermo-Oxidative Aging of the NBR/PAE/SWCNT Composites

**Figure 9:**
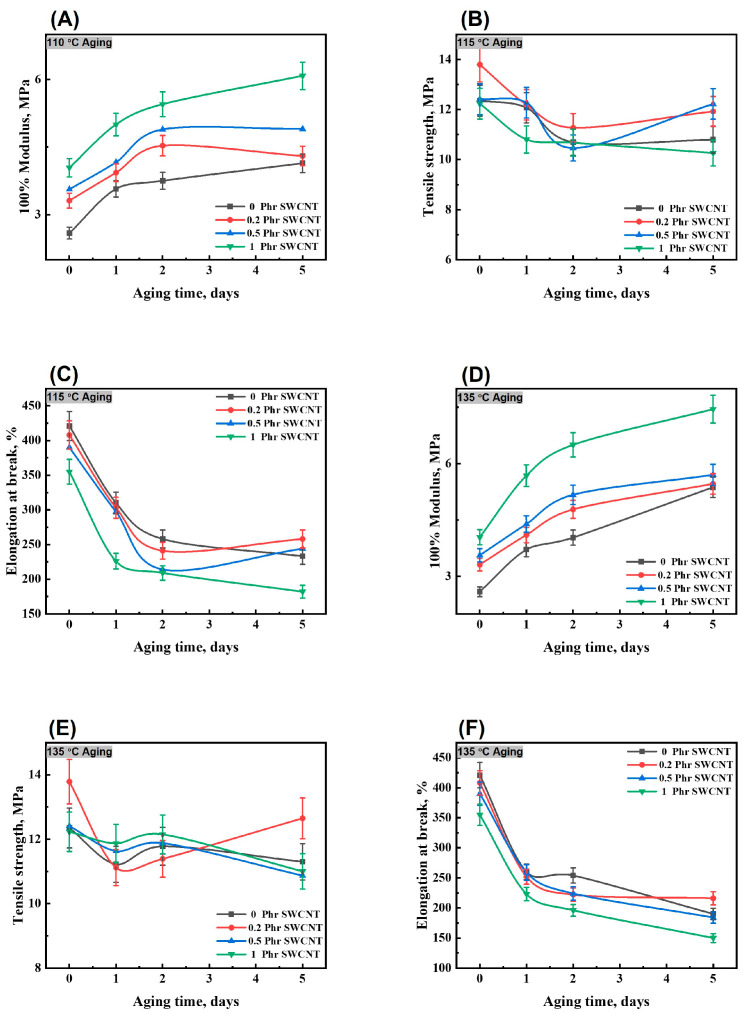
The 100% modulus, tensile strength, and elongation at break of NBR/PAE/SWCNT composites aging at (**A**–**C**) 115 °C and (**D**–**F**) 135 °C.

In previous work, it was found that NBR/PAE blends had better tensile strength retention at different aging temperatures than NBR and PAE, which has high potential for application [30]. In order to further optimize the properties, the effect of different SWCNT contents on the thermal-oxidative aging resistance of the NBR/PAE/SWCNT composites was investigated, as shown in Figure 9. It is clear that as the number of SWCNTs increased, the 100% constant tensile stress increased. As the aging process continued, the elongation at break decreased and 100% constant tensile stress gradually increased. This was attributed to the residual sulfide system not completing the reaction; in the aging process, the molecular chain continued to crosslink, resulting in a decrease in the elongation at break as well as an increase in the 100% constant tensile stress. However, it is interesting to note that the tensile strength of the NBR/PAE/SWCNT composites did not change significantly before and after aging. All of them had excellent tensile strength retention rates. This was attributed to the strong intermolecular interaction between NBR and PAE in the NBR/PAE/SWCNT composites, which was able to resist thermo-oxidative aging. Moreover, SWCNTs could interact with NBR/PAE, which may have led to the high tensile strength retention rates of the NBR/PAE/SWCNT composites under thermo-oxidative aging conditions.

### 3.6. Properties before and after Oil Aging and the Swelling Behavior of the NBR/PAE/SWCNT Composites

**Figure 10:**
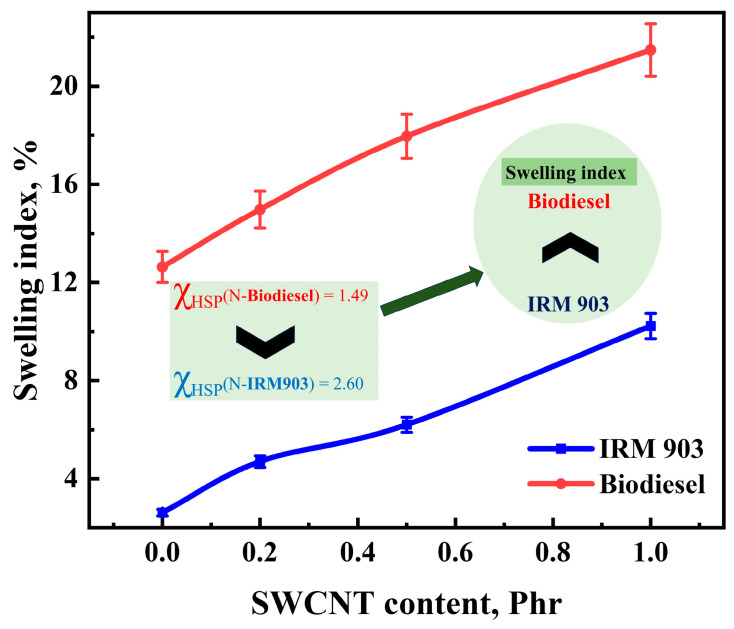
The swelling index of NBR/PAE/SWCNT composites immersed in biodiesel and IRM 903 at 115 °C.

**Figure 11:**
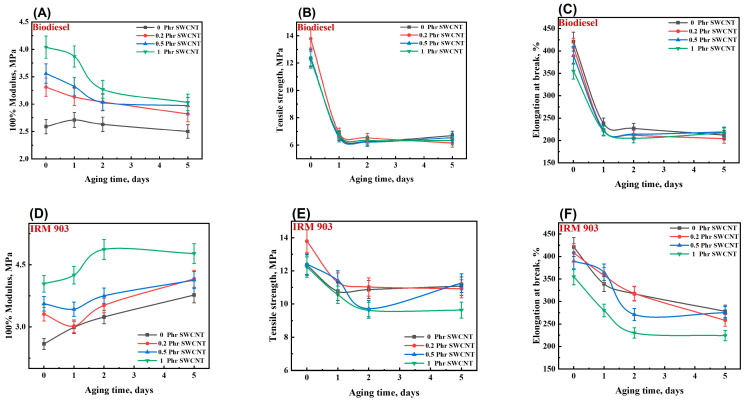
The tensile properties of NBR/PAE/SWCNT composites after immersion in biodiesel (**A**–**C**) and IRM 903 (**D**–**F**) for different times at 115 °C.

**Figure 12:**
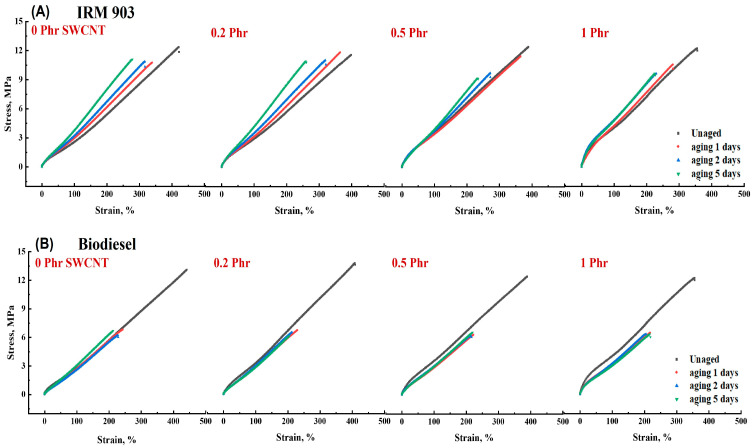
The tensile stress–strain curves of NBR/PAE/SWCNT composites after immersion in IRM 903 (**A**) and biodiesel (**B**) for different times at 115 °C.

To investigate the changes in NBR/PAE/SWCNT composite properties under oil aging, the NBR/PAE/SWCNT composites were immersed in IRM 903 as well as biodiesel under different aging conditions. After aging in oil for 5 days, the swelling index of the NBR/PAE/SWCNT composites immersed in biodiesel was significantly higher than that in IRM 903, as shown in Figure 10. According to a previous study [44], the HSP method was used to explore the swelling behavior of the NBR/PAE/SWCNT composites in different oils. Equation (4) calculated that the interaction parameters between the NBR/PAE/SWCNT composites and biodiesel were smaller than those with IRM 903 in the aging environment of 115 °C, leading to biodiesel being more likely to enter the composites. As shown in Figure 11 and Figure 12, the tensile strength and elongation at break of the composites after being immersed in the two oils for different aging times were dramatically reduced compared to the unaged samples, and the mechanical properties in biodiesel were more remarkably reduced. This was attributed to the fact that biodiesel entered the rubber more easily and destroyed the molecular chain structure of the rubber, resulting in a greater reduction in tensile strength and elongation at break. Interestingly, the 100% constant tensile stress of the composites immersed in biodiesel gradually decreased with increasing aging time, while the stress gradually increased in IRM 903 oil. After aging in oil at 115 °C, there was a residual sulfide system within the polymer, and the polymer continued to crosslink, increasing the constant elongation stress. Of course, the entry of small molecules into the oil also led to the destruction of the crosslinked polymer network, which resulted in a decrease in the constant tensile stress [30]. When aging in IRM 903 oil, fewer small molecules of oil entered the material compared to that immersed in biodiesel, and the increase in constant stress was due to the post-sulfurization process dominating the process. In contrast, the small molecules of oil had less effect on the disruption of the crosslinked network.

(4) $$\chi_{HSP}=\frac{V_{S}}{4RT}\times {Ra}^{2}$$

where *Vs* is the molar volume of the fuel; and *R* and *T* are the ideal gas constant and absolute temperature, respectively.

**Figure 13:**
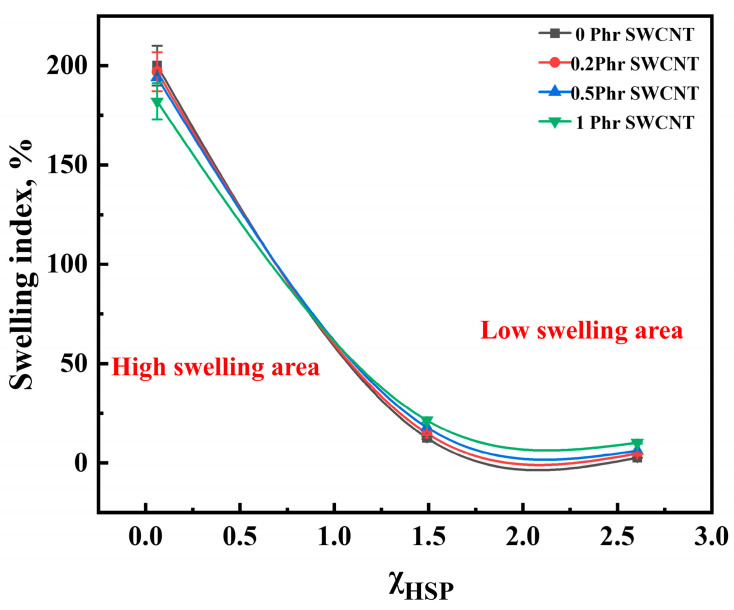
Correlation of the swelling index of NBR/PAE/SWCNT composites with χ_HSP_.

**Figure 14:**
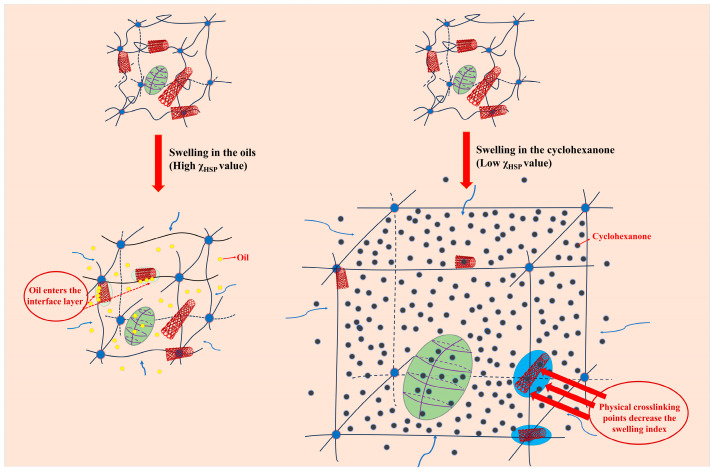
Schematic representation of the changes in the crosslinked network of NBR/PAE/SWCNT immersed in fluids with different interaction parameters.

Figure 13 shows that the swelling index increased with increasing SWCNT content after aging in two different oils for 5 days. Interestingly, after 5 days of immersion in cyclohexanone, the swelling index decreased with the increase in SWCNT content, showing a different regular variation. Based on this, the relationship between the swelling index and interaction parameters of the NBR/PAE/SWCNT composites with different SWCNT contents within different kinds of oils and solvents was investigated using the HSP method. In some solvents with small interaction parameters, the solvent entered the polymer network more easily, and the crosslinked network swelled to its maximum value. In turn, the interaction forces between SWCNTs and NBR/PAE molecular chains caused the network to retract, decreasing the swelling ratio with increasing SWCNT content. More interestingly, for some oils with large intermolecular interaction parameters, it was difficult for oil to enter the polymer network, and the swelling of the crosslinked network was not significant. Moreover, some weak interfaces between SWCNTs and the rubber were produced, leading to some oil molecules entering more easily. As a result, in some oils with large interaction parameters, the swelling index of the composites increased with increasing SWCNT content. Based on this, the variation in crosslinked polymer networks within liquids with different interaction parameters is plotted in Figure 14, which can better help us to understand the aging process.

## 4. Conclusions

In this work, the interactions between NBR/PAE and SWCNTs were investigated, and the high-temperature aging resistance as well as the oil aging resistance of the composites were studied. The vulcanization curves showed that the M_H_−M_L_ values of the composites gradually increased with the addition of SWCNTs, which was due to their large specific surface area and aspect ratio. The rheological properties of the composites were tested, and the composites storage moduli increased with the SWCNT increase. The 100% constant tensile stress and hardness both increased with the addition of SWCNTs, which was also related to the better reinforcing effect of SWCNTs. The decrease in the elongation at break might have resulted from the aggregation of SWCNTs. Combined with the equilibrium swelling test and analysis by the HSP method, it could be inferred that the interactions between SWCNTs and NBR/PAE formed a better interfacial layer. Thermo-oxidative aging tests at 115 and 135 °C showed that the addition of SWCNTs gave the material higher tensile strength retention and better thermo-oxidative aging properties. Considering that NBR composites are often applied in fields such as the automotive field, contact with oil is unavoidable. Therefore, the composites were considered when immersed in biodiesel at 115 °C and IRM 903 for aging performance testing. The tensile strength of the composites showed different degrees of reduction after aging in the two oils. The 100% constant tensile stress gradually decreased with time in biodiesel but gradually increased in IRM 903.

Interestingly, the swelling index of the rubber material gradually decreased with increasing SWCNT content after immersion in cyclohexanone for 5 days. However, the opposite phenomenon occurred after immersion in biodiesel as well as IRM 903. It could be calculated using the HSP method that the interaction parameters between the composite and cyclohexanone were much smaller than those with biodiesel and IRM 903, which led to a higher swelling index in cyclohexanone. The reason for the different swelling behaviors in different liquids could be deduced from the interaction parameters calculated using the HSP method and combined with the swelling index. The swelling volume of the rubber was regulated by controlling the swelling behavior. This is more favorable for some applications in which volume swelling is required. For example, some rubber sealings need swelling to a certain extent in order to achieve better sealing performance. In summary, this work provides good direction for the development and application of oil- and high-temperature-resistant products. Such products would be suitable for a variety of environments, providing protect against grease, fuel, and chemicals. Next, the abrasion resistance as well as chemical stability of the rubber in liquid environments need to be further explored.

## Figures and Tables

**Table 1:** Recipe for the NBR/PAE/SWCNT composites.

Sample/phr	1	2	3	4
NBR	100	100	100	100
PAE	20	20	20	20
N330	30	30	30	30
TMTD	0.5	0.5	0.5	0.5
DM	2	2	2	2
DMPPD	1	1	1	1
TMQ	0.5	0.5	0.5	0.5
ZnO	5	5	5	5
Stearic acid	1	1	1	1
Sulfur	1.5	1.5	1.5	1.5
SWCNT	0	0.2	0.5	1

**Table 2:** Curing properties of various NBR/PAE/SWCNT composites.

SWCNT Content (phr)	t_10_ (min)	t_90_ (min)	M_L_ (dNm)	M_H_ (dNm)
0	1.21	2.51	1.14	15.01
0.2	1.2	2.57	1.26	15.61
0.5	1.21	2.56	1.31	15.70
1	1.19	2.50	1.51	16.41

Note: phr = parts per hundred of rubber.

## Data Availability

The data presented in this study are available on request from the corresponding author.
